# Satellite Cells and Markers of Muscle Regeneration during Unloading and Reloading: Effects of Treatment with Resveratrol and Curcumin

**DOI:** 10.3390/nu12061870

**Published:** 2020-06-23

**Authors:** Laura Mañas-García, Maria Guitart, Xavier Duran, Esther Barreiro

**Affiliations:** 1 Pulmonology Department-Muscle Wasting and Cachexia in Chronic Respiratory Diseases and Lung Cancer Research Group, IMIM-Hospital del Mar, Parc de Salut Mar, Health and Experimental Sciences Department (CEXS), Universitat Pompeu Fabra (UPF), Barcelona Biomedical Research Park (PRBB), 08003 Barcelona, Spain; laura.manas@upf.edu (L.M.-G.); mguitart@imim.es (M.G.); 2 Centro de Investigación en Red de Enfermedades Respiratorias (CIBERES), Instituto de Salud Carlos III (ISCIII), 08003 Barcelona, Spain; 3 Scientific and Technical Department, Hospital del Mar-IMIM, 08003 Barcelona, Spain; xduran@imim.es

**Keywords:** muscle unloading, muscle reloading, sirtuin-1, muscle progenitor cells, activated satellite cells, quiescent satellite cells, muscle regeneration markers

## Abstract

We hypothesized that treatment with pharmacological agents known to increase sirtuin-1 activity (resveratrol and curcumin) may enhance muscle regeneration. In limb muscles of mice (C57BL/6J, 10 weeks) exposed to reloading for seven days following a seven-day period of hindlimb immobilization with/without curcumin or resveratrol treatment, progenitor muscle cell numbers (FACS), satellite cell subtypes (histology), early and late muscle regeneration markers, phenotype and morphometry, sirtuin-1 activity and content, and muscle function were assessed. Treatment with either resveratrol or curcumin in immobilized muscles elicited a significant improvement in numbers of progenitor, activated, quiescent, and total counts of muscle satellite cells, compared to non-treated animals. Treatment with either resveratrol or curcumin in reloaded muscles compared to non-treated mice induced a significant improvement in the CSA of both hybrid (curcumin) and fast-twitch fibers (resveratrol), sirtuin-1 activity (curcumin), sirtuin-1 content (resveratrol), and counts of progenitor muscle cells (resveratrol). Treatment with the pharmacological agents resveratrol and curcumin enhanced the numbers of satellite cells (muscle progenitor, quiescent, activated, and total satellite cells) in the unloaded limb muscles but not in the reloaded muscles. These findings have potential clinical implications as treatment with these phenolic compounds would predominantly be indicated during disuse muscle atrophy to enhance the muscle regeneration process.

## 1. Introduction

Disuse muscle atrophy is an important condition that is the result of progression of other chronic and acute diseases, such as cardiac and respiratory disorders; cancer; prolonged bed rest; and critical illness. Reduced physical activity leading to deconditioning is characterized by the loss of muscle mass and function in the affected muscles in patients [1,2,3,4,5] and in animal models [6,7,8]. Muscle atrophy resulting from deconditioning may also worsen disease prognosis in patients with chronic and acute diseases regardless of the underlying condition [9,10].

Several pathophysiological and biological mechanisms are involved in the loss of muscle mass and function characteristic of muscle atrophy. In this regard, a great many studies have previously demonstrated that markers of oxidative stress, inflammation, proteolysis, apoptosis, autophagy, and atrophy signaling pathways were upregulated in the atrophying muscles following periods of disuse in patients [11,12] and animal models [6,7,8]. Whether regenerative potential is altered in models of disuse muscle atrophy remains to be fully elucidated.

Skeletal muscles are formed as a result of the fusion of progenitor myoblasts during development. Postnatal muscle stem cells replace the muscle turnover resulting from the daily life activity of humans and animals [13]. Thus, regeneration of skeletal muscles is a tightly regulated process [13]. Muscle regeneration relies widely on the interaction between satellite cells and the microenvironment. Their numbers and subtypes may vary according to the underlying condition, such as during muscle atrophy [11,13,14], exposure to cigarette smoking [15], aging [16], and prolonged bed rest [17]. All these conditions may also take place simultaneously within the same individual and may interfere with the process of muscle regeneration in the patients.

Satellite cells are characterized by their heterogeneity, which leads to different functions within the skeletal muscle fibers. The satellite cell reservoir is composed by sublaminar cells that express paired box (Pax)-7 with no expression of myogenic factor (Myf)-5 marker [13]. Importantly, Pax-7+/Myf-5+ satellite cells are the ones that preferentially differentiate into muscle fibers, while Pax-7+/Myf-5- cells do not proliferate, representing the satellite cell reservoir of the muscles. The ability to express Myf-5 determines these two different subtypes of satellite cell populations within the skeletal muscles [13]. In line with this, Pax-7+/Myf-5- satellite cells are identified as the actual stem cells, whereas Pax-7+/Myf-5+ satellite cells are recognized as the committed myogenic progenitors [13].

Resveratrol elicits beneficial effects on tissues including skeletal muscles. It is a natural polyphenol, which is obtained from peanuts, red wine, grapes, and other plants. It is a very popular compound given its effects as a powerful antioxidant [18]. Resveratrol was also shown to improve the lifespan of different animals by attenuating oxidative stress, inflammation, and atherosclerosis [19,20,21,22]. Amelioration of injury was also shown in the gastrocnemius of rats in response to treatment with resveratrol [23]. Moreover, muscle regeneration also improved as a result of resveratrol treatment in mice [24,25].

Curcumin is another polyphenolic compound that is obtained from turmeric. Curcumin exerted beneficial effects on several tissues through different mechanisms. For instance, in mouse cells, myocardial-infarction-induced fibrosis was attenuated via sirtuin-1 activation [26]. Senescence of smooth muscle and endothelial cells also improved as a result of curcumin therapy via sirtuin-1 activity [27]. Inhibition of NF-kB elicited by curcumin was shown to attenuate muscle protein degradation in models, such as in sepsis [28,29] and during unloading in mice [30]. Moreover, muscle regeneration was also favored by treatment with the NF-kB inhibitor curcumin [31].

In models of unloading and reloading, it was shown that the acetylation statuses of the transcription factors fork-head box O (FoxO)1 and FoxO3 were relevant mediators of the process of muscle mass loss during unloading of the limb muscles in mice [6,7]. Interestingly, unloading of the gastrocnemius muscles elicited a decline in levels of histone deacetylase sirtuin-1 activity, and the activity level rose up to the control levels following a period of reloading of the hindlimb [6,7]. The beneficial effects observed in the gastrocnemius muscle in those studies were most likely due to the activity of sirtuin-1 enzyme on the transcription factors FoxO1 and FoxO3 [6,7]. Thus, it is plausible to conceive that enhancement of sirtuin-1 activity with compounds such as resveratrol and curcumin may prevent muscles from further muscle loss through attenuation of the activity of atrophy signaling. On the other hand, sirtuin-1 activity may also promote muscle repair and regeneration following disuse muscle atrophy [25].

On this basis, the current hypothesis was that treatment with pharmacological agents (resveratrol and curcumin) known to increase sirtuin-1 activity, among other functions, may enhance muscle regeneration as evaluated by the identification of biological players clearly involved in this process in the limb muscles of mice exposed to muscle reloading following a period of hindlimb unloading. Hence, the objectives were as follows, in the limb muscles of mice exposed to a seven-day period of unloading followed by another seven-day period of reloading treated with either resveratrol or curcumin: (1) progenitor muscle cell numbers (all limb muscles together) were identified using fluorescent-activated cell sorting (FACS), (2) subtypes of satellite cells in histological preparations of gastrocnemius muscle were counted, (3) markers of early and late muscle regeneration were analyzed, (4) fiber type composition and morphometry of the gastrocnemius muscle were assessed, (5) sirtuin-1 activity and content were explored, and (6) function of the limb muscles was also explored. The experimental model employed in the current investigation has previously been well validated [6,7,8].

## 2. Methods

### 2.1. Study Design and Animal Experiments

Female C57BL/6J mice (10 weeks old, weight ~20 g) were obtained from Harlan Interfauna Ibérica SL (Barcelona, Spain). Female mice were used for practical reasons as previous investigations in our group had also been conducted on this type of animal [6,7,8,30]. Mice were kept under pathogen-free conditions in the animal house facility at Barcelona Biomedical Research Park (PRBB), with a 12/12 h light–dark cycle.

**Figure 1:**
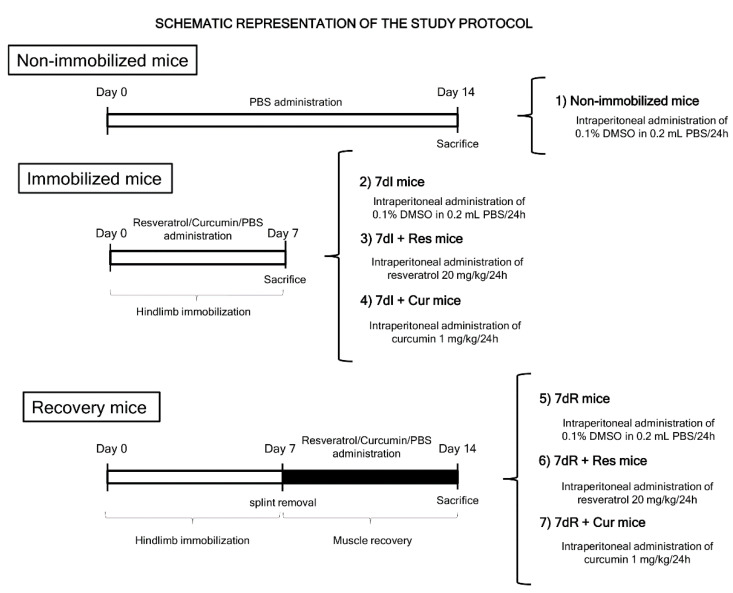
Graphical time-line representation of all the groups and treatments administered to the mice in the study. Definition of abbreviations: PBS, phosphate-buffered saline; DMSO, dimethyl sulfoxide; mL, millilitre; mg, milligram; kg, kilogram; h, hour; I, immobilization; R, recovery; Res, resveratrol; Cur, curcumin.

The entire study protocol is shown in Figure 1. Mice were exposed to unilateral hindlimb immobilization as previously described to reproduce a model of disuse muscle atrophy [6,7,8,32]. The time-points used in the current investigation have also been validated in previous studies conducted by our group [6,7,8]. Muscle damage was demonstrated in the limb muscles of the unloaded mice [7]. Reloading of the muscle for another 7-day period elicited an improvement in muscle damage [7].

The left hindlimb was shaved with clippers and was enveloped using surgical tape. The hindlimb was introduced into a 1.5-mL microcentrifuge tube with cover and bottom lids removed, while maintaining the foot in a plantar-flexed position to induce the maximal atrophy of the target limb muscle [6,7,8,32]. As the weight of the tube was approximately 0.6 g, it did not interfere with the usual mobility of the mice. The following groups of mice were studied (n = 10/group, Figure 1): (1) non-immobilized mice, (2) 7-day-immobilized mice (7dI, left hindlimb immobilized for seven consecutive days), (3) 7dI mice treated with resveratrol (7dI + Res, intraperitoneal administration, 20 mg/kg weight/24 h) [33,34], (4) 7dI mice treated with curcumin (7dI + Cur, intraperitoneal administration, 1 mg/kg weight/24 h) from day 0 to day 7 [35], (5) 7-day-recovery mice (7dR, left hindlimb immobilized for seven consecutive days, when the plastic splint was removed and the animals were moving free in their cages, in order to evaluate muscle recovery), (6) 7dR mice treated with resveratrol (7dR + Res, intraperitoneal administration, 20 mg/kg weight/24 h) from day 7 to day 14 [33,34], and (7) 7dR mice treated with curcumin (7dR + Cur, intraperitoneal administration, 1 mg/kg weight/24 h) from day 7 to day 14 [35].

The half-life of circulating curcumin was previously established to go from 15 to 60 min in animal models and patients [36,37] and from 30 to 60 min for resveratrol [38]. The rationale to administer resveratrol or curcumin intraperitoneally was to ensure that each animal received exactly the same dose of the drug every day. Administration of resveratrol or curcumin using other routes (oral, during food or water administration) would not allow us to ensure an identical dose for each mouse. Furthermore, intraperitoneal administration avoids absorption through the gastrointestinal tract and the first barrier of the hepatic metabolism, as generally happens in oral administration [39,40]. Accordingly, intraperitoneal injection was the selected route due to the fact that it gets into the circulation faster than other routes (oral gavage), to ensure the optimal absorption of the two compounds in view of its short bioavailability in plasma, estimated as 15–60 min in mice [36,37,38]. In order to control for a potential injection-induced stress response, all four groups (including the non-treated controls) of animals were injected intraperitoneally.

### 2.2. Ethics

All animal experiments were conducted in the animal facilities at PRBB. This was a controlled study designed in accordance with the ethical regulations on animal experimentation of the European Community Directive 2010/63/EU, Spanish Legislation (*Real Decreto* 53/2013, BOE 34/11370–11421) and the European Convention for the Protection of Vertebrate Animals Used for Experimental and Other Scientific Purposes (1986). All animal experiments were approved by the Animal Research Committee at PRBB. Ethical approval was obtained by the Animal Research Committee (Animal Welfare Department in Catalonia, Spain, EBP-13-1485).

### 2.3. In-Vivo Measurements in the Mice

In all the study animals, body weight and food intake were measured at every time point, and food and water were supplied ad libitum for the entire duration of the immobilization or recovery periods. In all mice, limb strength was determined on day 0 and right at the end of each immobilization or recovery time point (as described above) using a grip strength meter (Bioseb, Vitrolles Cedex, France) following previously published methodologies [6,7,8,30,41] in which grip strength was also the end-point parameter in the different experimental models. Grip strength was assessed in the four limbs at the same time in all mice. In all the animals, limb strength gain was calculated as the percentage of the measurements performed at the end of the study period with respect to the same measurements obtained at baseline (grip strength at the end of the study period − grip strength on day 0)/grip strength on day 0 × 100) [6,7].

### 2.4. Sacrifice and Sample Collection

Mice from all the experimental groups were sacrificed after the corresponding immobilization or recovery time cohorts (7 or 14 days). Each mouse was previously inoculated intraperitoneally with 0.1 mL sodium pentobarbital (60 mg/Kg). In all cases, the pedal and blink reflexes were evaluated in order to verify total anesthetic depth. Muscles were obtained from all the animals at the time of sacrifice. For isolation of muscle cell progenitor experiments, the following muscles were obtained from all the animals at the time of sacrifice: gastrocnemius, tibialis anterior (TA), extensor digitorum longus (EDL) and quadriceps femoris (QF, entire muscles in all cases). All study muscles from the hindlimb were pooled together by groups of two for each experimental condition: non-immobilized, 7dI and 7dR, with/without treatment with either resveratrol or curcumin. For immunofluorescence experiments, gastrocnemius muscle was obtained at the time of sacrifice in each experimental cohort and was embedded in optimum cutting temperature (OCT, Sakura Finetek, Torrance, CA, USA). For stem cell progenitor isolation experiments, the muscle samples were preserved in cold Dulbecco’s Modified Eagle’s Medium (DMEM) to be immediately processed as described below. For identification of several muscle regeneration markers, gastrocnemius muscle samples were snap-frozen in liquid nitrogen to be thereafter stored frozen at −80 °C to be further used.

### 2.5. Tissue Embedding

The gastrocnemius muscles of all study groups were fixed in 4% paraformaldehyde solution, pH 6.9 (EMD Millipore corporation, Billerica, MA, USA) and were embedded progressively with increasing concentrations of sucrose. They were subsequently embedded in tissue-tek OCT compound to be snap-frozen in 2-methyl-butane immersed in liquid nitrogen as previously described [42]. Ten-µm frozen sections were cut using a cryostat microtome (Leica CM3050S, Leica Biosystems, Wetzlar, Germany) at −20 °C and were mounted on glass slides.

### 2.6. Biological Analyses

*Muscle fiber type and morphometry.* Slow- and fast-twitch muscle fibers were identified using immunofluorescence procedures with anti-myosin heavy chain (MyHC) I and anti-MyHC II antibodies, respectively. Muscle cross-sections were air-dried for thirty minutes and were rinsed with phosphate-buffered saline (PBS) for another fifteen minutes. PBS was used to rinse sections in the different incubation steps. After rising, the sections were put in cold methanol for six more minutes. The sections were then boiled using a pressure cooker in 10 mM citrate buffer (pH 6.0) for twenty minutes and were then cooled down at room temperature for two hours. Subsequently, sections were incubated with mouse IgG blocking reagent (MOM, Vector Laboratories, Burlingame, CA, USA) for one hour, and in blocking solution (3% bovine serum albumin (BSA), 10% goat serum and 0.5% triton in PBS) for another hour. Afterwards, they were incubated overnight with the mouse monoclonal anti-MyHC I antibody (ab11083, Abcam) and anti-MyHC II antibody (ab51263, Abcam) prepared in blocking solution at 4 °C. Following incubation with the primary antibody and after rising with PBS, the sections were incubated with the corresponding secondary antibody and 4’,6-diamino-2-fenilindol (DAPI), which specifically stained deoxyribonucleic acid (DNA) allowing identification of all nuclei for one hour at room temperature: Alexa Fluor^®^ 488 AffiniPure goat anti-mouse IgG, Fcγ Subclass 1 Specific (Jackson Immunoresearch, West Grove, USA), which was also prepared in the blocking solution. The sections were mounted using 70% glycerol in 30% PBS. Myofibers positively stained with the anti-MyHC type I antibody or anti-MyHC type II were fluorescein isothiocyanate (FITC)-stained in green in two consecutives muscle cross-sections. The cross-sectional area (CSA), mean least diameter, and proportions of type I and type II, were assessed using a fluorescence microscope (x20 objective, Nikon Eclipse Ni, Nikon, Tokyo, Japan) coupled with an image-digitizing camera (Zyla 4.2 sCMOS camera, Andor, Belfast, UK) and the Image J software (National Institute of Health, available at http://rsb.info.nih.gov/ij/). In each muscle cross-section, at least 100 fibers were measured and counted, separately, from all study groups of mice. Fibers that were stained simultaneously for both anti-type I and anti-type II primary antibodies were identified as the hybrid fibers. They were all counted in the histological preparations of all the study groups.

*Satellite cell identification using immunofluorescence microscopy*. Immunofluorescence staining was used to detect satellite cells in both quiescent and activated states using specific antibodies (see below). Briefly, muscle cross-sections were air-dried for 30 min and were rinsed with PBS for another 15 min. PBS was used to rinse the sections among the different incubation steps. After rinsing, the sections were put in cold methanol for six more minutes. Then, the sections were boiled using a hot bath in 0.1 M citrate buffer (pH 6.0) for 12 min and were then blocked with 10% goat serum in PBS for two hours.

Subsequently, sections were incubated with MOM for 30 min. Afterwards, they were incubated overnight with a mixture of two antibodies: mouse monoclonal anti-Pax-7 antibody (Developmental Studies Hybridoma Bank, Iowa, IA, USA) and rabbit polyclonal anti-Myf-5 antibody (Aviva Systems Biology, San Diego, CA, USA), prepared in an antibody solution (1% goat serum dissolved in PBS, at 4 °C. Anti-Pax-7 antibody alone was used to detect quiescent satellite cells, while the mixture of anti-Pax-7 and anti-Myf-5 antibodies detected committed satellite cells [13]. The addition of quiescent and committed satellite cells corresponded to the total number of satellite cells. Following incubation with the primary antibodies and after rinsing with PBS, the sections were incubated at room temperature with the corresponding secondary antibodies for one hour: Alexa Fluor^®^ 488 AffiniPure goat anti-mouse IgG, Fcγ Subclass 1 Specific and Alexa Fluor^®^ plus 555 goat anti-rabbit IgG (H+L) (Thermo Fisher Scientific, Waltham, USA) also prepared in an antibody solution. Finally, the sections were mounted using the fluorescent mounting medium DAPI G-Fluoromount medium (Southern Biotech, Birmingham, AL, USA), which specifically marks DNA (allowing identification of all nuclei) in the muscle sections. A fluorescence microscope (×40 objective, Nikon Eclipse Ni, Nikon, Tokyo, Japan) coupled with a digitizing camera was used to identify and count the number of satellite cells (10 fields) in each study sample. Results were expressed as Pax-7+/Myf-5- (quiescent) satellite cells, Pax-7+/Myf-5+ (activated) satellite cells or the addition of both (as total satellite cells) to the total number of counted myonuclei in the 10 fields. Additionally, negative control experiments were carried out by omission of the primary antibodies and incubation of the muscle samples only with secondary antibody, to confirm the specificity of each antibody.

*Sirtuin-1 activity.* Briefly, frozen muscle samples from the gastrocnemius muscle of all study animals were homogenized in a buffer containing 50 mM 4-(2-hydroxyethyl)-1-piperazineethanesulfonic acid (HEPES), 150 mM NaCl, 100 mM NaF, 10 mM Na pyrophosphate, 5 mM ethylenediaminetetraacetic acid (EDTA), 0.5% Triton-X, and no protease inhibitors. Homogenates were centrifuged in a 4 mL buffer containing 30% sucrose, 10 mM Tris HCl (pH 7.5), 10 mM NaCl, and 3 mM MgCl_2_ at 4 °C and at 1300 *g* for 10 min. The pellets were washed with cold 10 mM Tris-HCl (pH 7.5) and 10 mM NaCl, to be subsequently centrifuged at 4 °C and at 1300 *g* for 10 min. The resultant pellets, which contained the nuclei, were resuspended in 200 µL of extraction buffer containing 50 mM HEPES potassium hydroxide (HEPES KOH, pH 7.5), 420 mM NaCl, 0.5 mM EDTA, 0.1 mM Ethylene glycol tetraacetic acid (EGTA) and 10% glycerol. The nuclei were subsequently sonicated in 15-s cycles. Afterwards, the sonicated nuclei remained on ice for 30 min. Following centrifugation at 4 °C and at 12,000 *g* for 10 min, the supernatants (crude nuclear extracts) were stored at −80 °C until further use [43,44]. Sirtuin-1 activity was evaluated using 50 µg of crude nuclear extracts from the gastrocnemius muscle of all experimental mice.

**Figure 2:**
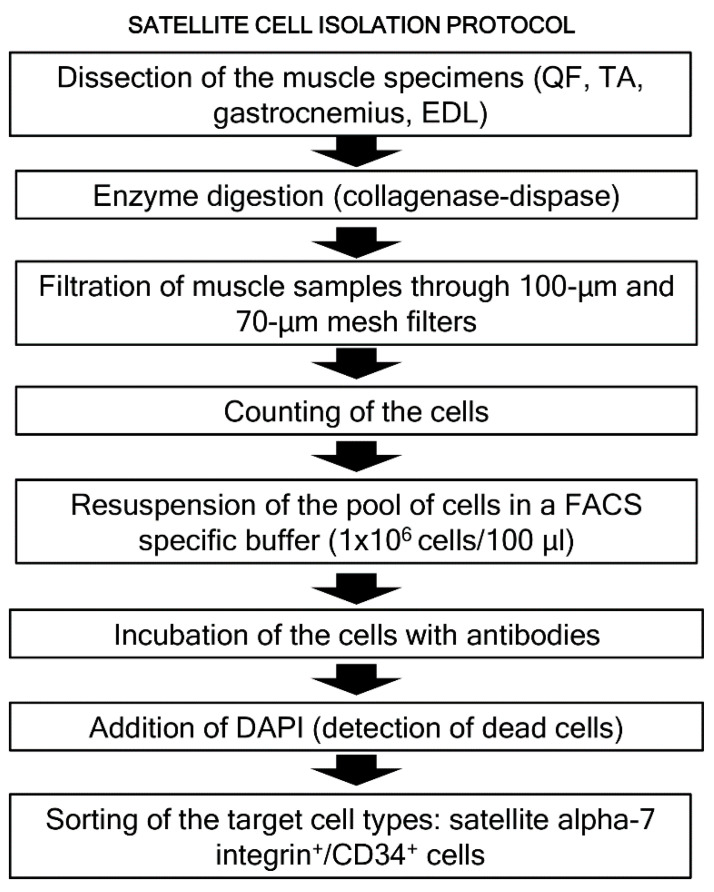
Representative flowchart corresponding to the isolation protocol of the satellite cells using FACS analyses in the mouse limb muscles. Each specific step is described in a box and the flow of the entire protocol is indicated by the dark thick arrows. Definition of abbreviations: QF, quadriceps femoris; TA, tibialis anterior; EDL, extensor digitorum longus; FACS, fluorescence-activated cell sorting; DAPI, 4’,6-diamino-2-fenilindol; α7, alpha-7 integrin.

*Satellite cell isolation using fluorescence-activated cell sorting (FACS).* A schematic representation of these methodologies is shown in Figure 2. Immediately after dissection, muscles were processed following a modified version of a previously described protocol [45]. Pools of gastrocnemius, TA, EDL and QF muscles were first minced with scissors and secondly with a razor blade (Electron Microscopy Science, Hatfield, PA, USA). The minced muscles were collected in a 50-mL tube containing 40 mL of cold DMEM to be subsequently washed to remove fat tissue. Enzymatic digestion with a DMEM media containing 2.5 U/mL collagenase (Serva Electrophoresis, Heidelberg, Germany) and 2.5 U/mL dispase (Sigma Aldrich, Sant Louis, MO, USA) was immediately performed at 37 °C in an agitation bath for 10 min.

The digestion procedure was repeated four times and the digested muscle solution was then filtered through a 100-µm mesh filter (Corning, New York, NY, USA). Immediately afterwards, the digestion was stopped by adding 2 volumes of 10% fetal bovine serum (FBS) in PBS, and the muscle solution was filtered through a 70-µm mesh filter (Corning). The filtered solution was centrifuged at 300× *g* for 5 min. The pellet was kept and the supernatant was centrifuged again in order to recover the maximum amount of cells. Finally, the two cell pellets were combined in a fresh tube to be re-suspended in FACS buffer ((1 mL PBS solution containing 2.5% goat serum (Sigma Aldrich)). The number of cells was counted using a Neubauer chamber.

Prior to incubation with antibodies, the cells were washed in 20-mL cold DMEM and centrifuged at 1700 rpm for 10 min to recover the cell pellet. Cells were resuspended in 1 × 10^6^ cell/100 µL FACS-specific buffer. All the cells were incubated with antibodies used to specifically identify the satellite cells: Phycoerythrin (PE)-conjugated anti-alpha-7 integrin (Ablab, Vancouver, Canada), a heterodimeric integral membrane protein critical for the modulation of cell–matrix interactions, and allophycocyanin (APC)-conjugated anti-CD34 (BD Pharmigen, San Jose, CA, USA), as a cell surface sialomucin (a mucopolysaccharide molecule containing sialic acid) with reported anti-adhesive, motile, and pre-proliferative properties for 30 min. Additionally, the cells from all study groups of mice were incubated at the same time (30 min) with specific antibodies to exclude other cell type populations that might also have been present in the muscle extracts, namely endothelial cells, leukocytes and hematopoietic cells: PE-cyanine7 (Cy7)-conjugated anti-CD31 (Biolegend, San Diego, CA, USA), a marker of endothelial cells, both anti-CD11b (Biolegend) and anti-CD45 (Biolegend) as markers of leukocytes, and anti-Sca-1 (Biolegend), a marker of hematopoietic stem cells. The excluded cell populations were named negative-lineage (Lin (−)).

Subsequently, all the study cells were incubated with DAPI in order to exclude the dead cells (cells DAPI+ exclusively) five minutes prior to the start of FACS analyses (FACS Aria II SORP, BD Biosciences, San Jose, CA, USA). Once DAPI+ cells and other cell types (endothelial, leukocytes, and hematopoietic) were excluded, the cells identified using FACS were small and of a round shape, indicating a relatively low complexity. Cells stained for PE-conjugated anti-alpha-7 integrin and APC-conjugated anti-CD34 were sorted and were named alpha-7 integrin^+^/CD34^+^ muscle progenitors. Muscle progenitor cells were expressed as the percentage of alpha-7 integrin^+^/CD34^+^ progenitors to the total grams of tissue.

*Ribonucleic acid (RNA) extraction.* Total RNA was first isolated from the gastrocnemius muscle of mice using Trizol reagent following the manufacturer’s protocol (Life Technologies, Carlsbad, CA, USA). Total RNA concentrations were determined spectrophotometrically using a NanoDrop 1000 (Thermo Scientific, Waltham, MA, USA).

*Procedures of messenger (mRNA) reverse transcription (RT).* A single RT was performed from which all the target genes of the study were analyzed. First-stranded complementary deoxyribonucleic acid (cDNA) was generated from mRNA using oligo(dT)_12–18_ primers and the Super-Script III reverse transcriptase following the manufacturer’s instructions (Life Technologies).

*Quantitative real-time *polymerase chain reaction* amplification (qRT-PCR).* TaqMan-based qPCR reactions were performed using the ABI PRISM 7900HT Sequence Detector System (Life Technologies, Carlsbad, CA, USA) together with commercially available gene expression assays. The probes corresponding to the following genes involved in muscle regeneration were tested: marker of cell proliferation Ki67 *(Ki67*) (Mm01278617_m1, Life Technologies). The housekeeping gene glyceraldehyde-3-phosphate dehydrogenase (*Gapdh*) (Mm99999915_g1, Life Technologies) served as the endogenous control for the mRNA gene [46,47]. Reactions were run in duplicate, and mRNA data were collected and subsequently analyzed using the Sequence Detection System relative quantification software, version 2.4 (Applied BioSystems), in which the comparative C_T_ method (2^-ΔΔCT^) for relative quantification was employed [48].

*Immunoblotting of 1D electrophoresis.* Protein levels of the different molecular markers analyzed in the study were explored by means of immunoblotting procedures as previously described [6,7,30]. Briefly, frozen muscle samples from the gastrocnemius muscle of all mouse experimental groups were homogenized in a buffer containing 50 mM HEPES, 150 mM NaCl, 100 mM NaF, 10 mM Na pyrophosphate, 5 mM EDTA, 0.5% Triton-X, 2 micrograms/mL leupeptin, 100 micrograms/mL phenylmethanesulfonyl fluoride (PMSF), 2 micrograms/mL aprotinin and 10 micrograms/mL pepstatin A. The entire procedure was conducted at 4 °C. Protein levels in crude homogenates were spectrophotometrically determined with the Bradford method using triplicates in each case and BSA as the standard (Bio-Rad protein reagent, Bio-Rad Inc., Hercules, CA, USA). The final protein concentration in each sample was calculated from at least two Bradford measurements that were almost identical. Equal amounts of total protein (ranging from 5 to 60 micrograms, depending on the antigen and antibody) from crude muscle homogenates were always loaded onto the gels, as well as identical sample volumes/lanes. For the purpose of comparison among the different groups of experimental and control rodents, muscle sample specimens were always run together and kept in the same order. Two independent sets of immunoblots were conducted and four fresh 10-well mini-gels were always simultaneously loaded for each of the antigens. Experiments were confirmed at least twice for all the antigens analyzed in the investigation.

Proteins were then separated by electrophoresis, transferred to polyvinylidene difluoride (PVDF) membranes, blocked with BSA and incubated overnight with selective primary antibodies. Protein levels of sirtuin-1 and myogenic markers Pax-7, myoD and myogein were identified in the gastrocnemius using specific primary antibodies: NAD-dependent protein deacetylase sirtuin-1 (anti-sirtuin-1 antibody, ProteinTech Group Inc., Rosemont, IL, USA), MyoD (anti-MyoD antibody, Santa Cruz Biotechnology, Santa Cruz, CA, USA), myogenin (anti-myogenin antibody, Santa Cruz Biotechnology), Pax-7 (anti-Pax-7 antibody; Abcam, Cambridge, UK) and GAPDH (anti-GAPDH antibody, Santa Cruz Biotechnology). Antigens from all samples were detected with horseradish peroxidase (HRP)-conjugated secondary antibodies and a chemiluminescence kit. For each of the antigens, samples from the different groups were always detected in the same picture under identical exposure times. The specificity of the different antibodies was confirmed by omission of the primary antibody and incubation of the membranes only with secondary antibodies.

PVDF membranes were scanned with the Alliance Q9 Advanced (Uvitec Cambridge, England, UK). Optical densities of specific bands were quantified using the ImageJ software (National Institute of Health, available at http://rsb.info.nih.gov/ij/). Final optical densities obtained in each specific group of mice corresponded to the mean values of the different samples (lanes) of each of the study antigens. In order to validate equal protein loading among the various lanes, the glycolytic enzyme GAPDH was used as the protein loading control in all the immunoblots [46,47].

### 2.7. Statistical Analysis

The Shapiro–Wilk test was used to test normality of the study variables. The results are presented as mean values (standard deviation). The variables of food intake and percentage changes in total body weight, limb strength, and muscle structure are represented in Table 1 and Table 2. The biological variables are represented in the figures. For each specific treatment (either resveratrol or curcumin), two-way analysis of variance (ANOVA) was performed using STATA (Software for Statistics and Data Science, StataCorp LLC, College Station, TX, USA) independently. For all the study variables, the following effects were analyzed: immobilization/recovery, treatment with either resveratrol or curcumin, and interaction between treatment and condition. Moreover, potential differences between two specific experimental groups were analyzed using post-hoc analysis contrast of marginal linear predictions: (1) comparisons between non-immobilized and immobilized mice, (2) comparisons between recovery and immobilized mice, (3) comparisons between treated and non-treated animals during the unloading period, and (4) comparisons between the treated and non-treated rodents during the recovery period. *p ≤* 0.05 was established as the level of significance.

## 3. Results

### 3.1. Physiological Characteristics of the Study Animals

*Non-immobilized versus immobilized conditions.* Compared with non-immobilized animals, in immobilized mice, total body and gastrocnemius weight and limb strength gain were significantly reduced, while food intake was not modified (Table 1).

*Reloading versus unloading conditions*. Compared with unloaded animals, in recovery mice, total body and gastrocnemius weight and limb strength gain significantly increased, while food intake was not modified (Table 1).

*Unloading with either resveratrol or curcumin versus unloading*. Compared to non-treated unloaded mice, in the gastrocnemius of 7dI + Res and 7dI + Cur mice, food intake, total body and gastrocnemius weight, and limb strength gain did not significantly differ (Table 1).

*Reloading with either resveratrol or curcumin versus reloading*. Compared to non-treated reloaded animals, in the gastrocnemius of 7dR + Res and 7dI + Cur mice, food intake, total body and gastrocnemius weight, and limb strength gain did not significantly differ (Table 1).

### 3.2. Structural Phenotypic Characteristics

*Non-immobilized versus immobilized conditions.* Compared with non-immobilized animals, in the gastrocnemius of immobilized mice, the cross-sectional area of both type I and type II muscle fibers significantly decreased, while the proportions of hybrid fibers increased (Table 2).

*Reloading versus unloading conditions*. Compared to non-treated unloading animals, in the gastrocnemius of the reloading mice, the CSA of type II fibers significantly increased (Table 2). Fiber type proportions of both slow- and fast-twitch and hybrid fibers did not significantly differ between 7dI and 7dR mice (Table 2). The CSA of slow-twitch and hybrid fibers did not differ between these two groups of animals (Table 2).

*Unloading with either resveratrol or curcumin versus unloading*. Compared to non-treated unloaded mice, in the gastrocnemius of 7dI + Res or 7dI + Cur mice, no significant differences were detected in either cross-sectional area or muscle fiber type proportions (Table 2).

*Reloading with either resveratrol or curcumin versus reloading*. Compared to non-treated reloaded animals, in the gastrocnemius of 7dR + Cur mice, the cross-sectional area of the hybrid fibers increased, as almost did the CSA of fast-twitch fibers in the 7dR + Res animals, while no significant differences were observed in the other parameters (Table 2).

### 3.3. Sirtuin-1 Content and Activity

**Figure 3:**
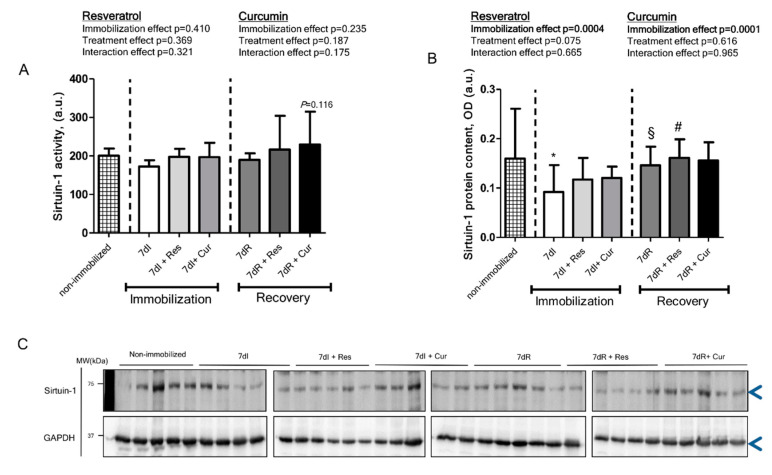
(**A**) Mean values and standard deviation of sirtuin-1 activity levels in the gastrocnemius muscle of the different study groups of mice, as measured by fluorescence in arbitrary units (a.u.). Definition of abbreviations: a.u., arbitrary units; I, immobilization; R, recovery; Res, resveratrol; Cur, Curcumin. *p* = 0.116: statistical significance between 7dR + Cur versus 7dR. The effect of immobilization and treatment and interaction effects are also indicated as actual *p* values for each variable. (**B**) Mean values and standard deviation of sirtuin-1 protein content in the gastrocnemius muscle of the different study groups of mice, as measured by optical densities in arbitrary units (OD, a.u.). Definition of abbreviations: OD, optical densities; a.u., arbitrary units; I, immobilization; R, recovery; Res, resveratrol; Cur, curcumin. Statistical significance is represented as follows: *, *p* < 0.05 between 7dI animals and non-immobilized mice; §, *p* < 0.05 between the 7dI and 7dR groups of animals; ^#^, *p* < 0.05 for any group of pharmacologically treated mice (resveratrol or curcumin) compared to their respective group (7dI or 7dR mice). The effect of immobilization and treatment and interaction effects are also indicated as actual *p* values for each variable. (**C**) Representative immunoblots of sirtuin-1 and GAPDH proteins in the gastrocnemius muscle of all study groups of mice. Arrowheads indicate the corresponding analyzed band. Definition of abbreviations: GAPDH, glyceraldehyde-3-phosphate dehydrogenase; MW, molecular weight; kDa, kilodalton; I, immobilization; R, recovery; Res, resveratrol; Cur, curcumin.

*Non-immobilized versus immobilized conditions.* Compared with non-immobilized animals, in the gastrocnemius of immobilized mice, sirtuin-1 activity did not differ, while sirtuin-1 protein levels significantly decreased (Figure 3A–C, respectively).

*Reloading versus unloading conditions*. Compared to non-treated unloading animals, in the muscles of the reloading mice, sirtuin-1 activity levels did not differ, while those of sirtuin-1 protein levels significantly increased (Figure 3A–C, respectively).

*Unloading with either resveratrol or curcumin versus unloading*. Compared to non-treated unloaded mice, in the gastrocnemius of 7dI + Res or 7dI + Cur mice, no significant differences were detected in either sirtuin-1 protein content or activity (Figure 3A–C, respectively).

*Reloading with either resveratrol or curcumin versus reloading*. Compared to non-treated reloaded animals, in the gastrocnemius of 7dR + Cur mice a significant rise in sirtuin-1 activity was almost (*p* = 0.116) observed, while sirtuin-1 protein levels showed a significant increase in 7dR + Res animals (Figure 3A–C, respectively).

### 3.4. Satellite Cell Counts

**Figure 4:**
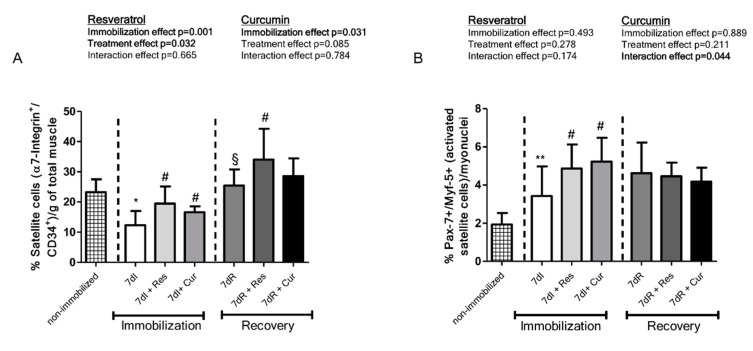

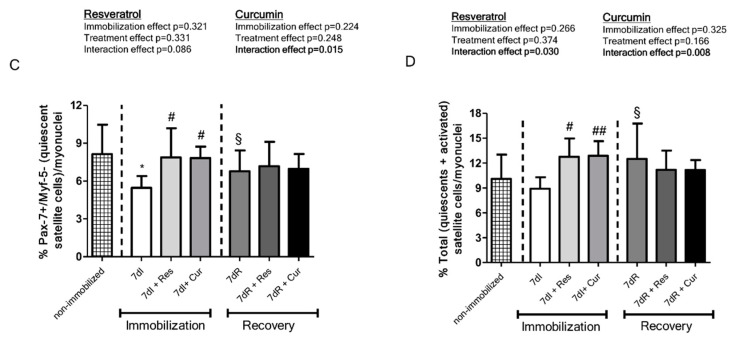
(**A**) Mean values and standard deviation of the percentage of satellite cells (alpha-7 integrin^+^/CD34^+^) measured by FACS analyses in the limb muscles of the different study groups of mice. Definition of abbreviations: α7, alpha-7 integrin; g, grams; I, immobilization; R, recovery; Res, resveratrol; Cur, curcumin. Statistical significance is represented as follows: *, *p* < 0.05 between the 7dI animals and non-immobilized mice; §, *p* < 0.05 between the 7dI and 7dR groups of animals; ^#^, *p* < 0.05 for any group of pharmacologically treated mice (resveratrol or curcumin) compared to their respective group (7dI or 7dR mice). The effect of immobilization and treatment and interaction effects are also indicated as actual *p* values for each variable. (**B**) Mean values and standard deviation of the percentage of activated satellite cell counts as identified by the number of Pax-7/Myf-5-positive cells in the gastrocnemius muscle of the different study groups of mice. *Definition of abbreviations*: Pax-7, paired box-7; Myf-5, myogenic factor 5; I, immobilization; R, recovery; Res, resveratrol; Cur, curcumin. Statistical significance is represented as follows: **, *p* < 0.01 between 7dI animals and non-immobilized mice; ^#^, *p* < 0.05 for any group of pharmacologically treated mice (resveratrol or curcumin) compared to their respective group (7dI or 7dR mice). The effect of immobilization and treatment and interaction effects are also indicated as actual *p* values for each variable. (**C**) Mean values and standard deviation of the percentage of quiescent satellite cell counts as identified by the number of Pax7-positive cells (Myf-5-negative) in the gastrocnemius muscle of the different study groups of mice. Definition of abbreviations: Pax-7, paired box-7; Myf-5, myogenic factor 5; I, immobilization; R, recovery; Res, resveratrol; Cur, curcumin. Statistical significance is represented as follows: *, *p* < 0.05 between 7dI animals and non-immobilized mice; §, *p* < 0.05 between the 7dI and 7dR groups of animals; ^#^, *p* < 0.05 for any group of pharmacologically treated mice (resveratrol or curcumin) compared to their respective group (7dI or 7dR mice). The effect of immobilization and treatment and interaction effects are also indicated as actual *p* values for each variable. (**D**) Mean values and standard deviation of the percentage of total satellite cell counts as identified by the number of quiescent and activated satellite cells in the gastrocnemius muscle of the different study groups of mice. Definition of abbreviations: I, immobilization; R, recovery; Res, resveratrol; Cur, curcumin. Statistical significance is represented as follows: §, *p* < 0.05 between the 7dI and 7dR groups of animals; ^#^, *p* < 0.05 and ^##^, *p* < 0.01 for any group of pharmacologically treated mice (resveratrol or curcumin) compared to their respective group (7dI or 7dR mice). The effect of immobilization and treatment and interaction effects are also indicated as actual *p* values for each variable.

**Figure 5:**
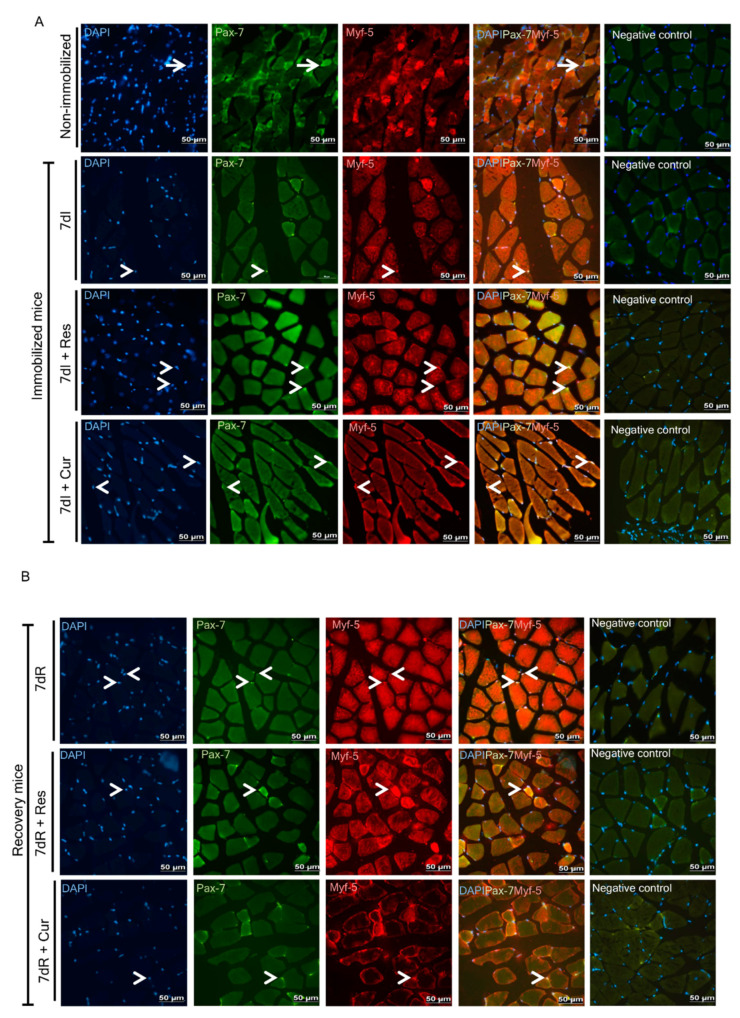
(**A**) Representative images of immunofluorescence staining of DAPI (left panels), Pax-7 (middle-left panels), Myf-5 (middle panels) and cells positively stained for both Pax-7 and Myf-5 markers (middle-right panels), and negative control (right panels) in the gastrocnemius muscle of the non-immobilized mice and immobilized mice with and without treatment (7dI, 7dI + Res and 7dI + Cur groups of mice). Thin arrows indicate Pax-7-positive cells, and arrowheads indicate double-stained nuclei for both Pax-7- and Myf-5-positive cells (activated satellite cells). Definition of abbreviations: Pax-7, paired box-7; Myf-5, myogenic factor 5; DAPI, 4’,6-diamino-2-fenilindol; I, immobilization; Res, resveratrol; Cur, curcumin. (**B**) Representative images of immunofluorescence staining of DAPI (left panels), Pax-7 (middle-left panels), Myf-5 ((middle panels) and cells positively stained for both Pax-7 and Myf-5 markers (middle-right panels) and negative control (right panels) in the gastrocnemius muscle of the recovery mice with and without treatment (7dR, 7dR + Res and 7dR + Cur groups of mice). Thin arrows indicate Pax-7-positive cells, and arrowheads indicate double-stained nuclei for both Pax-7- and Myf-5-positive cells (activated satellite cells). Definition of abbreviations: Pax-7, paired box-7; Myf-5, myogenic factor 5; DAPI, 4’,6-diamino-2-fenilindol; R, recovery; Res, resveratrol; Cur, curcumin.

*Non-immobilized versus immobilized conditions.* A significant decline in α7-integrin ^+^/CD34^+^ cells was detected in the limb muscles of the unloaded mice compared to non-immobilized animals (Figure 4A). Moreover, activated (Pax-7+ and Myf-5+) satellite cells increased in the gastrocnemius of the immobilized mice compared to the non-immobilized mice, while quiescent/regenerative potential (Pax-7+ and Myf-5-) cells decreased, and total numbers of satellite cells did not significantly differ in muscles between the two experimental groups (Figure 4B–D and Figure 5A, respectively).

*Reloading versus unloading conditions*. A significant increase in α7-integrin^+^/CD34^+^ cells was seen in the limb muscles of the reloading mice compared to immobilized animals (Figure 4A). Activated (Pax-7+ and Myf-5+) satellite cell numbers did not differ in the gastrocnemius of the recovery mice compared to unloaded muscles, while quiescent/regenerative potential (Pax-7+ and Myf-5-) cells and total numbers of satellite cells increased (Figure 4B–D and Figure 5A,B, respectively).

*Unloading with either resveratrol or curcumin versus unloading*. A significant increase in α7-integrin^+^/CD34^+^ cells was observed in the limb muscles of the unloading mice treated with either resveratrol or curcumin compared to immobilized animals (Figure 4A). A significant rise in activated (Pax-7+ and Myf-5+) satellite cells, quiescent/regenerative potential (Pax-7+ and Myf-5-) cells, and in total satellite cell numbers was observed in the gastrocnemius of the immobilized mice treated with either resveratrol or curcumin compared to immobilized animals (Figure 4B–D and Figure 5A, respectively).

*Reloading with either resveratrol or curcumin versus reloading*. A significant increase in α7-integrin^+^/CD34^+^ cells was observed in the limb muscles of the reloading mice treated with resveratrol compared to recovery animals, whereas curcumin elicited no significant modifications (Figure 4A). No significant differences in activated (Pax-7+ and Myf-5+) satellite cells, quiescent/regenerative potential (Pax-7+ and Myf-5-) cells, or total satellite cell numbers were detected in the gastrocnemius between recovery mice treated with either resveratrol or curcumin and non-treated recovery animals (Figure 4B–D and Figure 5B, respectively).

### 3.5. Myogenic Markers of Muscle Regeneration

**Figure 6:**
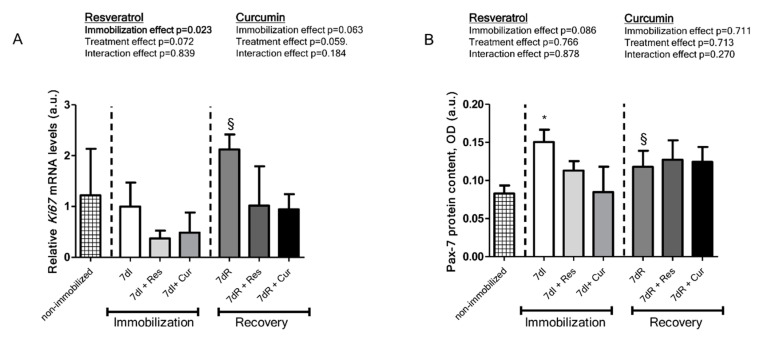

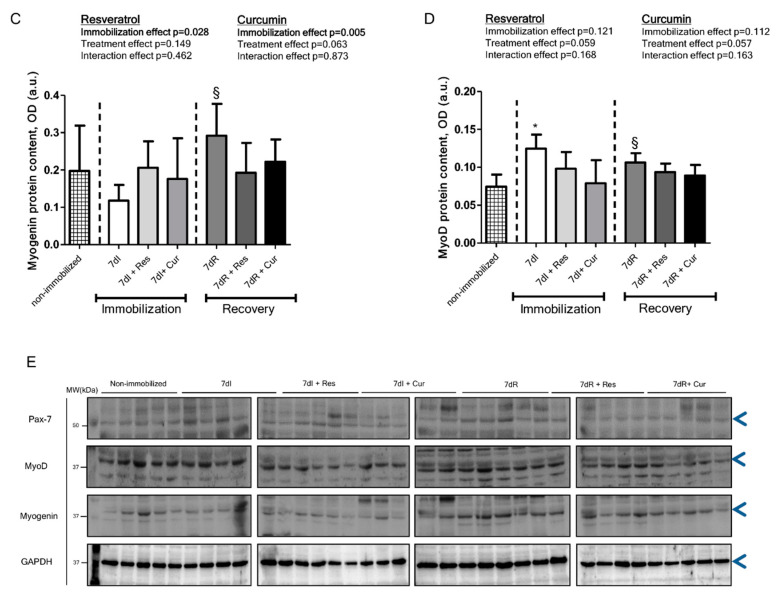
(**A**) Mean values and standard deviation of gene expression of *Ki67* in the gastrocnemius muscle of the different study groups of mice. Definition of abbreviations: Ki67, cell proliferation Ki-67; mRNA, messenger ribonucleic acid; a.u., arbitrary units; I, immobilization; R, recovery; Res, resveratrol; Cur, curcumin. Statistical significance is represented as follows: §, *p* < 0.05 between the 7dI and 7dR groups of animals. The effect of immobilization and treatment and interaction effects are also indicated as actual *p* values for each variable. (**B**) Mean values and standard deviation of Pax-7 protein content, as measured by optical densities in arbitrary units (OD, a.u.). Definition of abbreviations: OD, optical densities; a.u., arbitrary units; Pax-7, paired box-7; I, immobilization; R, recovery; Res, resveratrol; Cur, curcumin. Statistical significance is represented as follows: *, *p* < 0.05 between the 7dI animals and non-immobilized mice; §, *p* < 0.05 between the 7dI and 7dR groups of animals. The effect of immobilization and treatment and interaction effects are also indicated as actual *p* values for each variable. (**C**) Mean values and standard deviation of myogenin protein content, as measured by optical densities in arbitrary units (OD, a.u.). Definition of abbreviations: OD, optical densities; a.u., arbitrary units; I, immobilization; R, recovery; Res, resveratrol; Cur, curcumin. Statistical significance is represented as follows: §, *p* < 0.05 between the 7dI and 7dR groups of animals. The effect of immobilization and treatment and interaction effects are also indicated as actual *p* values for each variable. (**D**) Mean values and standard deviation of MyoD protein content, as measured by optical densities in arbitrary units (OD, a.u.). Definition of abbreviations: OD, optical densities; a.u., arbitrary units; MyoD, myogenic differentiation 1; I, immobilization; R, recovery; Res, resveratrol; Cur, curcumin. Statistical significance is represented as follows: *, *p* < 0.05 between 7dI animals and non-immobilized mice; §, *p* < 0.05 between the 7dI and 7dR groups of animals. The effect of immobilization and treatment and interaction effects are also indicated as actual *p* values for each variable. (**E**) Representative immunoblots of Pax-7, MyoD, myogenin and GAPDH proteins in the gastrocnemius muscle of all study groups of mice. Arrowheads indicate the corresponding analyzed band. Definition of abbreviations: Pax-7, paired box-7; MyoD, myogenic differentiation 1; GAPDH, glyceraldehyde-3-phosphate dehydrogenase; MW, molecular weight; kDa, kilodalton; I, immobilization; R, recovery; Res, resveratrol, Cur, curcumin.

*Non-immobilized versus immobilized conditions.* In the gastrocnemius muscle of immobilized rodents compared to non-immobilized mice, gene expression of *Ki67* did not differ (Figure 6A). In the gastrocnemius muscle of immobilized mice compared to non-immobilized animals, protein expression levels of Pax-7 significantly improved, those of myogenin did not differ, while those of MyoD increased (Figure 6B–E, respectively).

*Reloading versus unloading conditions*. In the gastrocnemius muscle of recovery mice compared to immobilized animals, gene expression of *Ki67* increased (Figure 6A). In the gastrocnemius muscle of recovery mice compared to immobilized animals, protein expression of Pax-7 significantly declined, myogenin increased, and MyoD significantly decreased (Figure 6B–E, respectively).

*Unloading with either resveratrol or curcumin versus unloading*. In the gastrocnemius of unloading mice treated with either resveratrol or curcumin compared to non-treated unloaded muscles, no differences were observed in *Ki67* expression levels (Figure 6A). In the gastrocnemius of unloading mice treated with either resveratrol or curcumin compared to non-treated unloaded muscles, protein expression of Pax-7, myogenin and MyoD did not differ (Figure 6B–E, respectively).

*Reloading with either resveratrol or curcumin versus reloading.* In the gastrocnemius of reloading mice treated with curcumin or resveratrol, compared to non-treated reloaded muscles, gene expression of *Ki67* did not differ (Figure 6A). In the gastrocnemius of reloading mice treated with either resveratrol or curcumin compared to non-treated reloaded muscles, protein expression of Pax-7, myogenin and MyoD did not differ (Figure 6B–E, respectively).

## 4. Discussion

The main findings of the current investigation were that in immobilized mice compared to non-immobilized mice total body and gastrocnemius weight, limb strength gain, CSA of both slow- and fast-twitch fibers, sirtuin-1 protein levels, numbers of muscle progenitor cells (α7-integrin^+^/CD34^+^), and numbers of quiescent (Pax-7+ and Myf-5-) satellite cells significantly decreased, while proportions of hybrid fibers, activated (Pax-7+ and Myf-5+) satellite cell counts, and both Pax-7 and MyoD protein levels significantly increased.

In the muscles of the reloading animals compared to unloaded mice, total body and gastrocnemius weight, limb strength gain, CSA of fast-twitch fibers, sirtuin-1 protein levels, numbers of α7-integrin^+^/CD34^+^ satellite cells, numbers of quiescent (Pax-7+ and Myf-5-) satellite cells, total counts of satellite cells, *Ki67* levels (gene expression), and myogenin protein levels significantly improved.

In muscles of the immobilized mice treated with either resveratrol or curcumin compared to non-treated immobilized animals, counts of satellite α7-integrin^+^/CD34^+^ cells, activated (Pax-7+ and Myf-5+) satellite cells, quiescent (Pax-7+ and Myf-5-) satellite cells, and the total counts of satellite cells improved.

In muscles of the reloaded animals treated with either resveratrol or curcumin compared to non-treated reloaded mice, the CSA of both hybrid (only in curcumin-treated mice) and fast-twitch fibers (only in resveratrol-treated animals), sirtuin-1 activity levels (only in curcumin-treated animals), sirtuin-1 protein levels (only in resveratrol-treated mice), and counts of α7-integrin^+^/CD34^+^ satellite cells (only in the resveratrol-treated mice) significantly increased.

Unloading of the limb muscles induced a significant decline in the numbers of muscle progenitor cells and quiescent satellite cells, while a rise in activated satellite cell counts was observed. These are interesting findings that put research forward on the impact of a seven-day period of unloading on the initial steps of the muscle regeneration process.

In different experimental models, treatment with resveratrol has been shown to improve muscle mass and phenotype as a result of sirtuin-1 activity [49] and attenuation of apoptosis [25]. Increased sirtuin-1 during reloading of previously unloaded muscles also induced an improvement in muscle mass and function [6]. In the current study, CSA of fast-twitch myofibers also improved in response to treatment with resveratrol during reloading of hindlimb muscles. These findings are in line with previous results obtained in our group (unpublished observations). On the other hand, curcumin administration during reloading also elicited an improvement in muscle phenotype and function through attenuation of apoptosis and proteolysis (ubiquitin–proteasome system) in experimental models [30,35]. Furthermore, NF-kB-dependent muscle wasting was also alleviated as a result of curcumin treatment in septic rats [28,29,50].

In the present study, treatment of immobilized mice with either resveratrol or curcumin induced a significant improvement in the numbers of muscle progenitor cells, quiescent, activated and total satellite cell counts in the limb muscles compared to non-treated immobilized animals. In line with this, it was previously shown [51] that sirtuin-1 maintained satellite cells in a stem-like state. This probably accounted for the rise in the numbers of quiescent and total satellite cells seen in the muscles of the unloaded mice treated with either curcumin or resveratrol. Furthermore, in trained elderly subjects treated with resveratrol, satellite cell numbers also increased in their limb muscles [52]. Collectively, these findings suggest that resveratrol and curcumin favor muscle regenerative potential following unloading.

Importantly, treatment with resveratrol was also shown to favor the process of muscle regeneration through several mechanisms. In fact, similar levels of muscle progenitor cells to those encountered in the current investigation were also reported in a former study [25]. Interestingly, a modest enhancement of myogenic precursor cell proliferation was seen in resveratrol-treated muscles following reloading [25]. Furthermore, recovery of muscle mass and of the size of fast-twitch fibers in the rat hindlimb muscles was also mediated by the action of proapoptotic proteins including cleaved caspase-3 [25]. Another mechanism whereby resveratrol may favor muscle regeneration was its ability to attenuate muscle damage (contusion model) in mice [24]. Furthermore, local and systemic markers of muscle regeneration were attenuated in response to resveratrol treatment in mice [24].

Specifically, muscle regenerative markers and recovery of normal tissue architecture was favored by the systemic administration of curcumin in mice in response to freeze injury [31]. The beneficial effects seen in muscles were due to blocking of NF-kB activity [31]. In a previous study from our group [30], curcumin also elicited an improvement in muscle phenotype and function via attenuation of NF-kB activity (lower levels of acetylation) in mice exposed to the same experimental conditions as in the present study. Taken together, these findings suggest that NF-kB is a probably a major regulator of the muscle regeneration process detected in the hindlimb muscles of mice in the present investigation.

Protein levels of early markers of muscle regeneration (Pax-7 and MyoD) significantly increased in the gastrocnemius of the immobilized mice compared to non-immobilized animals. These findings are in accordance with previous reports [8,17,53] and suggest that the process of muscle regeneration has been triggered. On the other hand, protein levels of the early markers Pax-7 and MyoD significantly declined in the gastrocnemius of the reloaded muscles, while those of the late marker myogenin significantly rose. These results are also consistent with previous reports in which late markers of muscle regeneration were upregulated during recovery in limb muscles [8,17,53].

Importantly, treatment with either curcumin or resveratrol in the reloading periods elicited an increase in sirtuin-1 activity and sirtuin-1 protein levels, while it did not induce any significant modifications in levels of any of the analyzed markers of muscle regeneration, cells or myogenic markers (ki67, pax-7, myoD, and myogenin) in the gastrocnemius of any of the reloaded animals. In keeping with this, a potential role of sirtuin-1 activity was established as a negative regulator of early myogenic markers (proliferation of muscle precursor cells) of muscle regeneration [54]. Another plausible explanation relies on the fact that the process of muscle regeneration would be almost entirely complete during the reloading phase, and treatment with any of the phenolic compounds would not be able to exert additional beneficial effects. Thus, treatment with either resveratrol or curcumin did not elicit any significant modification of the markers of muscle regeneration beyond the physiological effects elicited by the experimental conditions of unloading or reloading.

In line with this, treatment with resveratrol during recovery for two weeks of aged rats exposed to tail suspension also induced moderate therapeutic benefits as identified by muscle mass recovery and increased size of the fast-twitch fibers of limb muscles, probably resulting from the attenuation of apoptosis [25]. Another study [55] also demonstrated that the beneficial effects induced by resveratrol on the limb muscles of type I diabetic mice was not dependent on sirtuin-1 activity. In that investigation, mitochondrial membrane potential was restored in muscle fibers without the interference of sirtuin-1 activity in the mouse muscles [55].

## 5. Conclusions

Unloading of the limb muscles triggered a program of muscle regeneration characterized by the activation of satellite cells and the upregulation of early myogenic factors. Treatment with the pharmacological agents resveratrol and curcumin enhanced the numbers of the identified subtypes of satellite cells (muscle progenitor, quiescent, activated, and total satellite cells) in the unloaded limb muscles but not in the reloaded muscles. These findings have potential clinical implications as treatment with these phenolic compounds would predominantly be indicated during disuse muscle atrophy to enhance the muscle regeneration process. Treatment with either curcumin or resveratrol would not elicit as many beneficial effects during reloading.

## Figures and Tables

**Table 1:** Physiological parameters in all experimental groups of mice.

	**Non-Immobilized** **(*N* = 10)**	**7dI** **(*N* = 10)**	**7dI + Res** **(*N* = 10)**	**7dI + Cur** **(*N* = 10)**	**7dR** **(*N* = 10)**		**7dR + Res** **(*N* = 10)**		**7dR + Cur** **(*N* = 10)**
**Food intake (g/24 h)**	3.15 (0.10)	3.10 (0.03)	3.17 (0.13)	3.02 (0.56)	3.08 (0.47)		3.11 (0.17)		3.48 (0.65)
**Total body weight gain (%)**	+7.28 (2.02)	−1.60 (1.82), ***	+0.36 (1.49)	−0.73 (1.20)	+3.23 (1.45), §§		+6.40 (1.92)		+5.94 (1.30)
**Gastrocnemius weight (g)**	0.115 (0.003)	0.085 (0.05), *	0.091 (0.007)	0.089 (0.003)	0.097 (0.004), §		0.099 (0.004)		0.097 (0.006)
**Limb strength gain (%)**	+11.44 (5.10)	−5.45 (1.12), *	−0.61 (1.45)	−2.88 (1.81)	+7.00 (2.39), §§		+7.71 (2.08)		+7.60 (1.99)
	**Resveratrol**			**Curcumin**					
	**Immobilization effect** ***p*-value**	**Treatment effect** ***p*-value**	**Interaction effect** ***p*-value**	**Immobilization effect** ***p*-value**		**Treatment effect** ***p*-value**		**Interaction effect** ***p*-value**	
**Food intake (g/24 h)**	0.919	0.454	0.750	0.640		0.426		0.308	
**Total body weight gain (%)**	**0.0001**	0.052	0.621	**0.0001**		0.126		0.423	
**Gastrocnemius weight (g)**	**0.0001**	0.078	0.274	**0.0001**		0.270		0.270	
**Limb strength gain (%)**	**0.048**	0.547	0.637	**0.0171**		0.763		0.890	

Variables are presented as mean (standard deviation). Definition of abbreviations: I, immobilization; R, recovery; g, grams. Statistical significance is represented as follows: *, *p* < 0.05 and ***, *p* < 0.001 between 7dI animals and non-immobilized mice; §, *p* < 0.05 and §§, *p* < 0.01 between the 7dI and 7dR groups of animals. The effect of immobilization and treatment and interaction effects are also indicated as actual *p* values for each variable; *p* < 0.05 and *p* < 0.001 are presented in bold.

**Table 2:** Structural characteristics of the gastrocnemius muscle in the study groups.

	**Non-Immobilized** **(N = 10)**	**7dI** **(N = 10)**	**7dI + Res** **(N = 10)**	**7dI + Cur** **(N = 10)**	**7dR** **(N = 10)**		**7dR + Res** **(N = 10)**		**7dR + Cur** **(N = 10)**
**Muscle fiber type, %**									
Type I fibers	15.37 (2.99)	16.93 (2.88)	14.68 (2.56)	18.27 (6.00)	16.25 (7.23)		15.21 (8.28)		14.95 (4.84)
Type II fibers	81.96 (5.74)	82.94 (3.09)	85.31 (2.56)	81.72 (6.00)	83.74 (7.23)		84.78 (8.28)		85.04 (4.84)
**Muscle fiber size (CSA)**									
Cross-sectional area, type I fibers	1246.64 (129.93)	886.56 (115.34), **	982.45 (114.50)	843.59 (133.10)	923.18 (108.64)		1010.70 (111.03)		1035.55 (117.76)
Cross-sectional area, type II fibers	1256.52 (105.47)	914.31 (134.64), *	1002.68 (114.12)	948.48 (115.00)	1079.80 (119.22), §		1256.11 (127.88) (*p* = 0.075)		1117.72 (120.27)
Muscle hybrid fiber, %	0.66 (0.10)	3.55 (1.80), **	3.14 (1.05)	2.60 (0.67)	3.34 (1.72)		2.11 (1.10)		2.04 (0.99)
Cross-sectional area, hybrid fibers	1014.60 (86.57)	908.03 (150.27)	916.61 (187.89)	787.31 (88.64)	751.68 (167.49)		857.90 (128.07)		971.03 (167.32), #
	**Resveratrol**			**Curcumin**					
	**Immobilization effect** ***p*-value**	**Treatment effect** ***p*-value**	**Interaction effect** ***p*-value**	**Immobilization effect** ***p*-value**		**Treatment effect** ***p*-value**		**Interaction effect** ***p*-value**	
Type I fibers, %	0.890	0.275	0.850	0.195		0.726		0.908	
Type II fibers, %	0.890	0.275	0.850	0.325		0.926		0.423	
Cross-sectional area, type I fibers	0.750	0.156	0.832	0.210		0.770		0.470	
Cross-sectional area, type II fibers	**0.010**	0.147	0.713	**0.0140**		0.563		0.911	
Muscle hybrid fiber, %	0.810	0.352	0.990	0.455		0.587		0.674	
Cross-sectional area, hybrid fibers	0.515	0.694	0.576	0.283		0.621		**0.0187**	

Variables are presented as mean (standard deviation). Definition of abbreviations: I, immobilization; R, recovery; Res, resveratrol; Cur, curcumin; CSA, cross-sectional area. Statistical significance is represented as follows: *, *p* < 0.05 and **, *p* < 0.01 between 7dI animals and non-immobilized mice; §, *p* < 0.05 between the 7dI and 7dR groups of animals; #, *p* < 0.05 for any group of pharmacologically treated mice (resveratrol or curcumin) compared to their respective experimental group (7dI or 7dR mice). *p* = 0.075: statistical significance between 7dR + Res versus 7dR. The effect of immobilization and treatment and interaction effects are also indicated as actual *p* values for each variable; *p* < 0.05 and *p* < 0.01 are presented in bold.

## Editorial Support

None to declare.

## References

[1] Shrikrishna D., Patel M., Tanner R., Seymour J.M., Connolly B., Puthucheary Z., Walsh S.L., Bloch S., Sidhu P., Hart N. (2012). Quadriceps wasting and physical inactivity in patients with COPD. Eur. Respir. J..

[2] Schmidt S.F., Rohm M., Herzig S., Diaz M.B. (2018). Cancer Cachexia: More Than Skeletal Muscle Wasting. Trends Cancer.

[3] Barreiro E. (2019). Impact of Physical Activity and Exercise on Chronic Obstructive Pulmonary Disease Phenotypes: The Relevance of Muscle Adaptation. Arch. Bronconeumol..

[4] Gea J., Pascual S., Castro-Acosta A., Hernández-Carcereny C., Castelo R., Márquez-Martín E., Montón C., Palou A., Faner R., Furlong L.I. (2019). The Biomepoc Project: Personalized Biomarkers and Clinical Profiles in Chronic Obstructive Pulmonary Disease. Archivos de Bronconeumología (Engl. Ed.).

[5] Gea J., Martínez-Llorens J. (2019). Muscle Dysfunction in Chronic Obstructive Pulmonary Disease: Latest Developments. Archivos de Bronconeumología (Engl. Ed.).

[6] Chacon-Cabrera A., Gea J., Barreiro E. (2016). Short- and Long-Term Hindlimb Immobilization and Reloading: Profile of Epigenetic Events in Gastrocnemius. J. Cell. Physiol..

[7] Chacon-Cabrera A., Lund-Palau H., Gea J., Barreiro E. (2016). Time-Course of Muscle Mass Loss, Damage, and Proteolysis in Gastrocnemius following Unloading and Reloading: Implications in Chronic Diseases. PLoS ONE.

[8] Guitart M., Lloreta J., García L.M., Barreiro E. (2018). Muscle regeneration potential and satellite cell activation profile during recovery following hindlimb immobilization in mice. J. Cell. Physiol..

[9] Marquis K., Debigaré R., Lacasse Y., Leblanc P., Jobin J., Carrier G., Maltais F. (2002). Midthigh Muscle Cross-Sectional Area Is a Better Predictor of Mortality than Body Mass Index in Patients with Chronic Obstructive Pulmonary Disease. Am. J. Respir. Crit. Care Med..

[10] Maltais F., Decramer M., Casaburi R., Barreiro E., Burelle Y., Debigare R., Dekhuijzen P.N.R., Franssen F., Gayan-Ramirez G., Gea J. (2014). An official American Thoracic Society/European Respiratory Society statement: Update on limb muscle dysfunction in chronic obstructive pulmonary disease. Am. J. Respir. Crit. Care Med..

[11] Wall B.T., Dirks M.L., Snijders T., Senden J.M.G., Dolmans J., Van Loon L. (2013). Substantial skeletal muscle loss occurs during only 5 days of disuse. Acta Physiol..

[12] Simpson J.A., Labugger R., Collier C., Brison R.J., Iscoe S., Van Eyk J.E. (2005). Fast and Slow Skeletal Troponin I in Serum from Patients with Various Skeletal Muscle Disorders: A Pilot Study. Clin. Chem..

[13] Kuang S., Kuroda K., Le Grand F., Rudnicki M.A. (2007). Asymmetric Self-Renewal and Commitment of Satellite Stem Cells in Muscle. Cell.

[14] Mozdziak P., Pulvermacher P.M., Schultz E. (2001). Muscle regeneration during hindlimb unloading results in a reduction in muscle size after reloading. J. Appl. Physiol..

[15] Nogueira L., Trisko B.M., Lima-Rosa F.L., Jackson J., Lund-Palau H., Yamaguchi M., Breen E.C. (2018). Cigarette smoke directly impairs skeletal muscle function through capillary regression and altered myofibre calcium kinetics in mice fatigue resistance and myofibre calcium handling, and these changes ultimately affect contractile efficiency of locomotor muscles independent of a change in lung function. J. Physiol..

[16] Suetta C., Frandsen U., Mackey A.L., Jensen L., Hvid L.G., Bayer M.L., Petersson S.J., Schrøder H.D., Andersen J.L., Aagaard P. (2013). Ageing is associated with diminished muscle re-growth and myogenic precursor cell expansion early after immobility-induced atrophy in human skeletal muscle. J. Physiol..

[17] Arentson-Lantz E.J., English K.L., Paddon-Jones D., Fry C.S. (2016). Fourteen days of bed rest induces a decline in satellite cell content and robust atrophy of skeletal muscle fibers in middle-aged adults. J. Appl. Physiol..

[18] Jackson J.R., Ryan M.J., Hao Y., E Alway S. (2010). Mediation of endogenous antioxidant enzymes and apoptotic signaling by resveratrol following muscle disuse in the gastrocnemius muscles of young and old rats. Am. J. Physiol. Integr. Comp. Physiol..

[19] Donnelly L., Newton R., Kennedy G.E., Fenwick P.S., Leung R.H.F., Ito K., Russell R.E., Barnes P.J. (2004). Anti-inflammatory effects of resveratrol in lung epithelial cells: Molecular mechanisms. Am. J. Physiol. Cell. Mol. Physiol..

[20] Jarolim S., Millen J., Heeren G., Laun P., Goldfarb D.S., Breitenbach M. (2004). A novel assay for replicative lifespan in Saccharomyces cerevisiae. FEMS Yeast Res..

[21] Jiang Q., Cheng X., Cui Y., Xia Q., Yan X., Zhang M., Lan G., Liu J., Shan T., Huang Y. (2019). Resveratrol regulates skeletal muscle fibers switching through the AdipoR1-AMPK-PGC-1α pathway. Food Funct..

[22] Zhu W., Chen S., Li Z., Zhao X., Li W., Sun Y., Zhang Z., Ling W., Feng X. (2014). Effects and mechanisms of resveratrol on the amelioration of oxidative stress and hepatic steatosis in KKAy mice. Nutr. Metab..

[23] Feng Y., He Z., Mao C., Shui X., Cai L. (2019). Therapeutic Effects of Resveratrol Liposome on Muscle Injury in Rats. Med. Sci. Monit..

[24] Hsu Y.-J., Ho C.-S., Lee M.-C., Ho C.-S., Huang C.-C., Kan N.-W. (2020). Protective Effects of Resveratrol Supplementation on Contusion Induced Muscle Injury. Int. J. Med. Sci..

[25] Bennett B.T., Mohamed J.S., E Alway S. (2013). Effects of Resveratrol on the Recovery of Muscle Mass Following Disuse in the Plantaris Muscle of Aged Rats. PLoS ONE.

[26] Ji X., Xiao J., Sheng X., Zhang X., Guo M. (2016). Curcumin protects against myocardial infarction-induced cardiac fibrosis via SIRT1 activation in vivo and in vitro. Drug Des. Dev. Ther..

[27] Grabowska W., Suszek M., Wnuk M., Lewinska A., Wasiak E., Sikora E., Bielak-Zmijewska A. (2016). Curcumin elevates sirtuin level but does not postpone in vitro senescence of human cells building the vasculature. Oncotarget.

[28] Poylin V., Fareed M.U., O’Neal P., Alamdari N., Reilly N., Menconi M., Hasselgren P.-O. (2008). The NF-kappaB inhibitor curcumin blocks sepsis-induced muscle proteolysis. Mediat. Inflamm..

[29] Jin B., Li Y.-P. (2007). Curcumin prevents lipopolysaccharide-induced atrogin-1/MAFbx upregulation and muscle mass loss. J. Cell. Biochem..

[30] Mañas-García L., Bargalló N., Gea J., Barreiro E. (2020). Muscle Phenotype, Proteolysis, and Atrophy Signaling During Reloading in Mice: Effects of Curcumin on the Gastrocnemius. Nutrients.

[31] Thaloor D., Miller K.J., Gephart J., Mitchell P.O., Pavlath G.K. (1999). Systemic administration of the NF-κB inhibitor curcumin stimulates muscle regeneration after traumatic injury. Am. J. Physiol. Cell Physiol..

[32] Lang S.M., Kazi A.A., Hong-Brown L., Lang C.H. (2012). Delayed Recovery of Skeletal Muscle Mass following Hindlimb Immobilization in mTOR Heterozygous Mice. PLoS ONE.

[33] Park S.-J., Ahmad F., Philp A., Baar K., Williams T., Luo H., Ke H., Rehmann H., Taussig R., Brown A.L. (2012). Resveratrol Ameliorates Aging-Related Metabolic Phenotypes by Inhibiting cAMP Phosphodiesterases. Cell.

[34] Chang C.-C., Yang M.-H., Tung H.-C., Chang C.-Y., Tsai Y.-L., Huang J.-P., Yen T.-H., Hung L.-M. (2014). Resveratrol exhibits differential protective effects on fast- and slow-twitch muscles in streptozotocin-induced diabetic rats *J*. Diabetes.

[35] Vazeille E., Slimani L., Claustre A., Magne H., Labas R., Bechet D., Taillandier D., Dardevet M., Astruc T., Attaix D. (2012). Curcumin treatment prevents increased proteasome and apoptosome activities in rat skeletal muscle during reloading and improves subsequent recovery. J. Nutr. Biochem..

[36] Anand P., Kunnumakkara A.B., Newman R.A., Aggarwal B.B. (2007). Bioavailability of Curcumin: Problems and Promises. Mol. Pharm..

[37] Gutierres V.O., Campos M.L., Arcaro C.A., Assis R.P., Baldan-Cimatti H.M., Peccinini R.G., Paula-Gomes S., Kettelhut I.C., Baviera A.M., Brunetti I.L. (2015). Curcumin Pharmacokinetic and Pharmacodynamic Evidences in Streptozotocin-Diabetic Rats Support the Antidiabetic Activity to Be via Metabolite(s). Evid. Based Complement. Altern. Med..

[38] Baltaci S.B., Mogulkoc R., Baltaci A.K. (2016). Resveratrol and exercise (review). Biomed. Rep..

[39] Liu H.-W., Su Y.-K., Bamodu O.A., Hueng D.-Y., Lee W.-H., Huang C.-C., Deng L., Hsiao M., Chien M., Yeh C.-T. (2018). The Disruption of the β-Catenin/TCF-1/STAT3 Signaling Axis by 4-Acetylantroquinonol B Inhibits the Tumorigenesis and Cancer Stem-Cell-Like Properties of Glioblastoma Cells, In Vitro and In Vivo. Cancers.

[40] Turner P.V., Brabb T., Pekow C., Vasbinder M.A. (2011). Administration of Substances to Laboratory Animals: Routes of Administration and Factors to Consider. J. Am. Assoc. Lab. Anim. Sci..

[41] Barreiro E., Puig-Vilanova E., Marin-Corral J., Chacon-Cabrera A., Salazar-Degracia A., Mateu X., Maestu L.P., Garcia-Arumi E., Andreu A.L., Molina L. (2015). Therapeutic Approaches in Mitochondrial Dysfunction, Proteolysis, and Structural Alterations of Diaphragm and Gastrocnemius in Rats with Chronic Heart Failure. J. Cell. Physiol..

[42] Barthel L.K., Raymond P.A. (1990). Improved method for obtaining 3-microns cryosections for immunocytochemistry. J. Histochem. Cytochem..

[43] Wilkie G.S., Schirmer E.C. (2008). Purification of Nuclei and Preparation of Nuclear Envelopes from Skeletal Muscle. Adv. Struct. Saf. Stud..

[44] Vilà L., Elias I., Roca C., Ribera A., Ferre T., Casellas A., Lage R., Franckhauser S., Bosch F. (2014). AAV8-mediated Sirt1 gene transfer to the liver prevents high carbohydrate diet-induced nonalcoholic fatty liver disease. Mol. Ther. Methods Clin. Dev..

[45] Pasut A., Oleynik P., Rudnicki M.A. (2011). Isolation of Muscle Stem Cells by Fluorescence Activated Cell Sorting Cytometry. Adv. Struct. Saf. Stud..

[46] Kuang J., Yan X., Genders A., Granata C., Bishop D.J. (2018). An overview of technical considerations when using quantitative real-time PCR analysis of gene expression in human exercise research. PLoS ONE.

[47] Touchberry C.D., Wacker M.J., Richmond S.R., Whitman S.A., Godard M.P. (2006). Age-Related Changes in Relative Expression of Real-Time PCR Housekeeping Genes in Human Skeletal Muscle. J. Biomol. Tech..

[48] Livak K.J., Schmittgen T.D. (2001). Analysis of relative gene expression data using real-time quantitative PCR and the 2^−ΔΔC*T*^ method. Methods.

[49] Kuno A., Tanno M., Horio Y. (2015). The effects of resveratrol and SIRT1 activation on dystrophic cardiomyopathy. Ann. N. Y. Acad. Sci..

[50] He J., Xie H., Wu S. (2016). Dietary supplementation of curcumin alleviates NF-κB-dependent skeletal muscle wasting in rat. Endocr. Metab. Immune Disord. Drug Targets.

[51] Ryall J., Dell’Orso S., Derfoul A., Juan A., Zare H., Feng X., Clermont D., Koulnis M., Gutierrez-Cruz G., Fulco M. (2015). The NAD (+)-dependent SIRT1 deacetylase translates a metabolic switch into regulatory epigenetics in skeletal muscle stem cells. Cell Stem Cell.

[52] Alway S.E., McCrory J.L., Kearcher K., Vickers A., Frear B., Gilleland D.L., Bonner D.E., Thomas J.M., Donley D.A., Lively M.W. (2017). Resveratrol Enhances Exercise-Induced Cellular and Functional Adaptations of Skeletal Muscle in Older Men and Women. J. Gerontol. Ser. A Boil. Sci. Med. Sci..

[53] Mackey A.L., Kjaer M., Charifi N., Henriksson J., Bojsen-Møller J., Holm L., Kadi F. (2009). Assessment of satellite cell number and activity status in human skeletal muscle biopsies. Muscle Nerve.

[54] Pardo P.S., Boriek A.M. (2011). The physiological roles of Sirt1 in skeletal muscle. Aging.

[55] Jeong J., Conboy M.J., Conboy I.M. (2013). Sirt1-Independent Rescue of Muscle Regeneration by Resveratrol in Type I Diabetes. J. Diabetes Metab..

